# Novel and extendable genotyping system for human respiratory syncytial virus based on whole‐genome sequence analysis

**DOI:** 10.1111/irv.12936

**Published:** 2021-12-10

**Authors:** Jiani Chen, Xueting Qiu, Vasanthi Avadhanula, Samuel S. Shepard, Do‐Kyun Kim, James Hixson, Pedro A. Piedra, Justin Bahl

**Affiliations:** ^1^ Center for Ecology of Infectious Diseases, Institute of Bioinformatics University of Georgia Athens GA USA; ^2^ Department of Infectious Disease University of Georgia Athens GA USA; ^3^ Center for Communicable Disease Dynamics, Department of Epidemiology Harvard T.H. Chan School of Public Health Boston MA USA; ^4^ Department of Molecular Virology & Microbiology Baylor College of Medicine Houston TX USA; ^5^ Influenza Division Centers for Disease Control and Prevention Atlanta GA USA; ^6^ Department of Epidemiology, Human Genetics & Environmental Sciences, School of Public Health University of Texas Health Science Center Houston TX USA; ^7^ Department of Epidemiology and Biostatistics University of Georgia Athens GA USA

**Keywords:** genotype, genotypic classification, label software, phylogenetic analysis

## Abstract

**Background:**

Human respiratory syncytial virus (RSV) is one of the leading causes of respiratory infections, especially in infants and young children. Previous RSV sequencing studies have primarily focused on partial sequencing of G gene (200–300 nucleotides) for genotype characterization or diagnostics. However, the genotype assignment with G gene has not recapitulated the phylogenetic signal of other genes, and there is no consensus on RSV genotype definition.

**Methods:**

We conducted maximum likelihood phylogenetic analysis with 10 RSV individual genes and whole‐genome sequence (WGS) that are published in GenBank. RSV genotypes were determined by using phylogenetic analysis and pair‐wise node distances.

**Results:**

In this study, we first statistically examined the phylogenetic incongruence, rate variation for each RSV gene sequence and WGS. We then proposed a new RSV genotyping system based on a comparative analysis of WGS and the temporal distribution of strains. We also provide an RSV classification tool to perform RSV genotype assignment and a publicly accessible up‐to‐date instance of Nextstrain where the phylogenetic relationship of all genotypes can be explored.

**Conclusions:**

This revised RSV genotyping system will provide important information for disease surveillance, epidemiology, and vaccine development.

## 1 INTRODUCTION

Human respiratory syncytial virus (RSV) is a major cause of acute lower respiratory tract infection worldwide in infants and young children (<5 years of age), as well as in the elderly and patients who are immunocompromised.^1^ Despite the clinical significance and the burden of RSV infection, we lack an understanding of the patterns of virus emergence, evolution, and spread. Phylogenetic studies of RSV evolution are in need, especially on a global scale due to the limited availability of whole‐genome sequence (WGS) data and strongly asynchronous sampling in time and space.^2^

RSV is an enveloped virus with a negative‐sense, single‐stranded, non‐segmented RNA genome of ~15,200 nucleotides (nt) in length and belongs to the family *Pneumoviridae*. This genome encodes for 11 proteins, including the polymerase (L), nucleocapsid (N), phosphoprotein (P), transcriptional regulators (M2–1 and M2–2), matrix (M), small hydrophobic surface protein (SH), non‐structural proteins (NS1, NS2), and two major surface glycoproteins (F and G).^3^ This virus has been classified as subtype A (RSV‐A) or subtype B (RSV‐B) according to reactivity with monoclonal antibodies.^4^ Both subtypes typically co‐circulate during epidemic seasons. Within the RSV‐A and RSV‐B subtypes, different genotypes have been further classified mainly based on genetic differences in the second hypervariable region (HR) located at the G glycoprotein.^5^ Like other respiratory viruses, RSV has diverse circulation patterns. Several genotypes can co‐circulate within the same community, whereas novel RSV genotypes with high genomic diversity may arise and potentially replace the previous dominant genotypes.^6^ Fourteen genotypes among RSV‐A (GA1–7, SAA1, CB‐A, NA1–4, and ON1) and 24 genotypes in RSV‐B (GB1‐GB5, SAB1‐SAB4, URU1–2, BA1–12, and CB1) have been identified.^1^ The most notable genotype change observed in recent years is the emergence of RSV‐A (ON) and RSV‐B (BA) strains with a partial duplication of the distal third of the G gene and have since become the dominant strains in many regions.^7^, ^8^

With the emergence of novel genotypes, a potential association of RSV genotype with disease severity or geographic and temporal restriction of virus circulation has been reported^9^.^10^ Moreover, RSV genetic diversity has been considered as an important factor that allows for reinfections to occur and needs to be considered in vaccine development.^9^ Therefore, a genotyping system that could reflect RSV genetic diversity is needed. Previous RSV sequencing has largely focused on complete^11^ or partial G gene (200–300 nt)^3^, ^12^ for genotype characterization or diagnostics. However, the evolutionary signals from other gene regions should also be taken into account as the phylogeny inferred from other genes might conflict with the phylogeny inferred from complete or partial G gene alone. Furthermore, the novel identified G gene duplication signature should be considered as a single evolutionary event, whereas current widely used phylogenetic models only account for residue substitution events. In addition, most of the current RSV genotyping methods are based on the pairwise distance (*p‐distance*) matrix by specifying a cutoff value below which individuals are assigned to the same cluster.^13^, ^14^ It is important to note that several factors affect *p‐distance* calculation, including the length of the sequences and the number of sequences used in the analysis. The *p‐distance* defined genotype system also needs to be updated frequently due to the accumulated viral diversity within genotypes over an increased circulation period, and new genotypes are likely to be defined within the previously defined genotypes. Despite the need to easily recognize RSV genotypes for molecular epidemiology, vaccine design, and control efforts, the delineating criteria of RSV genotypes are not agreed upon.^11^, ^14^, ^15^, ^16^ There is a need for a robust system to define RSV genotypes and to resolve inconsistencies present in the literature arising from previous genotyping methods.

Our study proposed a novel and extendable RSV genotyping system based on a more complete RSV phylogeny. After evaluating the phylogeny inferred from different RSV gene datasets, we concluded that the WGS is the most informative and desirable dataset for RSV genotyping purpose. We categorized RSV into two classification levels, the genotype and subclade, mainly with phylogenetic analysis and detection year of sequences, which provides a convenient and sustainable way to refer to the emergence of RSV strains.

## 2 METHODS

### 2.1 Data management

RSV sequences from human clinical samples were retrieved from NCBI's GenBank nucleotide database using the search term “HRSV” on April 20, 2019. For all these sequences, metadata including the collection date, isolation country, and previously determined genotype were extracted from the GenBank records using program gbmunge (https://github.com/sdwfrost/gbmunge). For spatial distribution estimation, the isolation country of each sequence has been further grouped into six WHO regions.^17^ These sequences were then assigned to a subtype based on the best match in a nucleotide BLAST alignment against RSV‐A and RSV‐B reference sequences (GenBank acc. no: NC_038235.1 and NC_001781.1). Sequence alignment was generated using mafft.v7 ^18^ and subsequently manually edited in Seqotron to accommodate the open reading frames of all genes.^19^ The following inclusion and exclusion criteria were applied: (a) each sequence must include collection date (at least year); (b) the sequence length for each gene region should be longer than 70% length in the reference sequence; (c) sequences with unexpected spurious frame‐shift indel in the alignment were removed; (d) the recombinant sequences that could interfere the phylogenetic inferences, were identified using the detection methods RDP, GENECONV, MAXCHI, BOOTSCAN, and SISCAN that are implemented in the Recombination Detection Program RDP4 and were removed^20^ (Table S1). The final datasets consisted of 860 RSV‐A sequences and 591 RSV‐B sequences, respectively. The open reading frames of 10 RSV genes (NS1, NS2, N, P, M, SH, G, F, M2, and L) were extracted, and the WGS was generated with a concatenation of each gene.

### 2.2 Phylogenetic inference

Phylogenetic analysis for different gene datasets was conducted with the maximum likelihood (ML) approach using raxml v8.0,^21^ which has the advantage of partitioned analysis. We applied the autoMRE option embedded in raxml for an efficient convergence of bootstrapping process, where the bootstrapping value is one of the criteria for the genotype assignment. We implemented an indel coding method to code gene duplication and deletion region of the G gene as separate binary partitions. The rest of the nucleotides were set as a separate partition with the GTR + Gamma substitution model. The temporal signal of the WGS datasets was diagnosed using tempest, and temporal outliers were removed.^22^

Tree topology tests for the phylogenies inferred from different RSV gene datasets were performed using iq‐tree with the Shimodaira–Hasegawa (SH) test and approximately unbiased (AU) test.^23^ The evolutionary rates for different genes were estimated using the program treetime.^24^

### 2.3 Genotypes and subclades assignment for RSV

We aim to classify RSV strains into two levels in our analysis. The groups of strains that have potential to circulate are further defined as subclades under the classification level of genotypes. The genotype assignment is based on pair‐wise node distance. Pair‐wise node distances, which are the distances between the most common ancestors of groups of sequences in a phylogeny, were calculated between all nodes in a phylogenetic tree based on the alignment of the RSV WGSs, using the rrphylo v2.5.7 package.^25^ We further employed time (years) of detection for sequences within each clade as another criterion for subclade assignment, which is calculated by the oldest and the latest date of the sequences within clade using personal scripts in R v4.0.2. The R package ape v2.3^26^ was used to define genotypes and subclades, whereas visualizations were created with ggtree v1.16.0.^27^

### 2.4 RSV genotype classification tool

RSV genotype classification tool was built with Lineage Assignment by Extended Learning (label https://wonder.cdc.gov/amd/flu/label/) pipeline,^28^ which rapidly determines cladistic information for sequences using support vector machines (SVMs) without the need for time‐consuming sequence alignment, phylogenetic tree construction, or manual annotation. Sequences with an annotated genotype or subclade were used to create a training data library. Training data for each genotype were subsampled in an ad hoc manner using pda v1.0.3.^29^ (Table S2). The classification module (available at https://github.com/JianiC/rsv-genotype/tree/master/LABEL/RSV) was then built with training sequences and the custom scripts that are implemented in the label program. Both WGS and partial sequences of RSV can be automated to a genotype or subclade, and no further information is required.

## 3 RESULTS

### 3.1 Phylogenetic analysis with different RSV gene datasets

**FIGURE 1:**
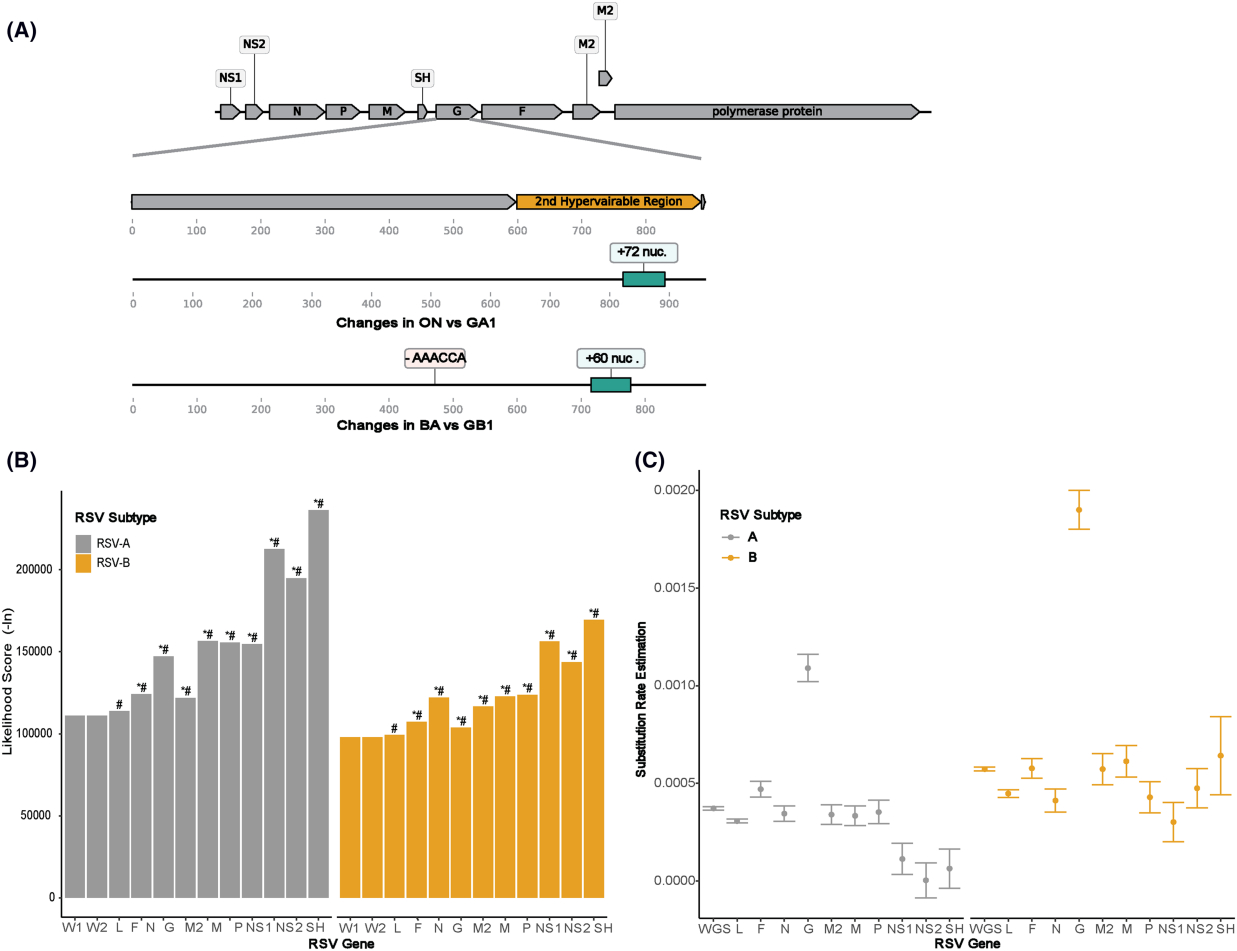
Scheme of respiratory syncytial virus (RSV) genome and comparison of RSV phylogenies inferred from different gene datasets. (A) RSV genome organization with G gene indels. (B) Likelihood scores of phylogenies inferred from different gene sequences given to the whole‐genome sequence (WGS) dataset. W1, the phylogeny inferred from WGS with G gene indels implemented as a single evolutionary event; W2, the phylogeny inferred from WGS with G gene indels implemented as multiple substitution events. *P < 0.005 in Shimodaira–Hasegawa (SH) test compared with W1; ^#^ *P* < 0.005 in approximately unbiased (AU) test compared with W1. (C) Comparison of evolutionary rates that are estimated from different gene datasets. Error bars indicate the confidence intervals of the estimation

We first characterized the indels of RSV sequences in our dataset. In addition to the previously defined RSV‐A genotype ON with a 72‐nt duplication in the second HR of G gene and RSV‐B genotype BA with a 60‐nt duplication in a similar region, we also observed a 6‐nt deletion in the recent circulating RSV‐B strains at the G gene region (Figure 1A).

The RSV phylogenies were constructed for each gene as well as WGS using the ML approach and the G gene duplication and deletion region have been further coded as a separate binary partition in our analysis. We also scored the likelihood of phylogenies inferred from different gene datasets given by the WGS dataset to compare the topologies of different phylogenetic trees. The SH and AU tests suggested the phylogenetic trees inferred from individual RSV genes have significantly different likelihood scores compared with WGS, and we did not observe the significant difference with the phylogeny where G gene indels were simply considered as multiple substitution events (Figure 1B). The tree topology differences inferred from the WGS and individual gene sequences (L, G, and F that have close likelihood scores with that of WGS) have been further demonstrated using a tanglegram approach as demonstrated in Figure S1. The mean nucleotide substitution rates for RSV‐A and RSV‐B estimated from WGS are 3.72 × 10^−4^ and 5.73 × 10^−4^ substitutions/site/year, respectively. The rate estimation of the G gene was approximately 2.5‐fold faster than other genes (Figure 1C). Overall, our results indicate the phylogenetic analysis based on WGS can provide more valuable insight on RSV genetic diversity and evolution.

### 3.2 Novel RSV genotype system with WGS

The following criteria were used to build a standardized RSV genotyping system:

1. Genotype and subclade designations are based on the phylogeny derived from WGS:
  - A supported monophyletic clade is defined with ≥70% bootstrap value at the node.
  - Genotypes are assigned by a maximal pair‐wise node distance within the clade, 0.018 for RSV‐A and 0.024 for RSV‐B (we simulate genotype assignment with different cutoff values in Figure S2).
  - Each genotype must contain at least 3 isolates.
  - Under the genotype level, the supported monophyletic clades with a detection time of at least 5 years are assigned as subclades. Time (years) of detection for each monophyletic clade is calculated by the oldest and the latest year of isolation of the sequences.
2. The genotype or subclade containing the oldest isolated sequence for RSV‐A or RSV‐B is named as A.1 or B.1, following the same logic for naming the next genotype or subclade.

**FIGURE 2:**
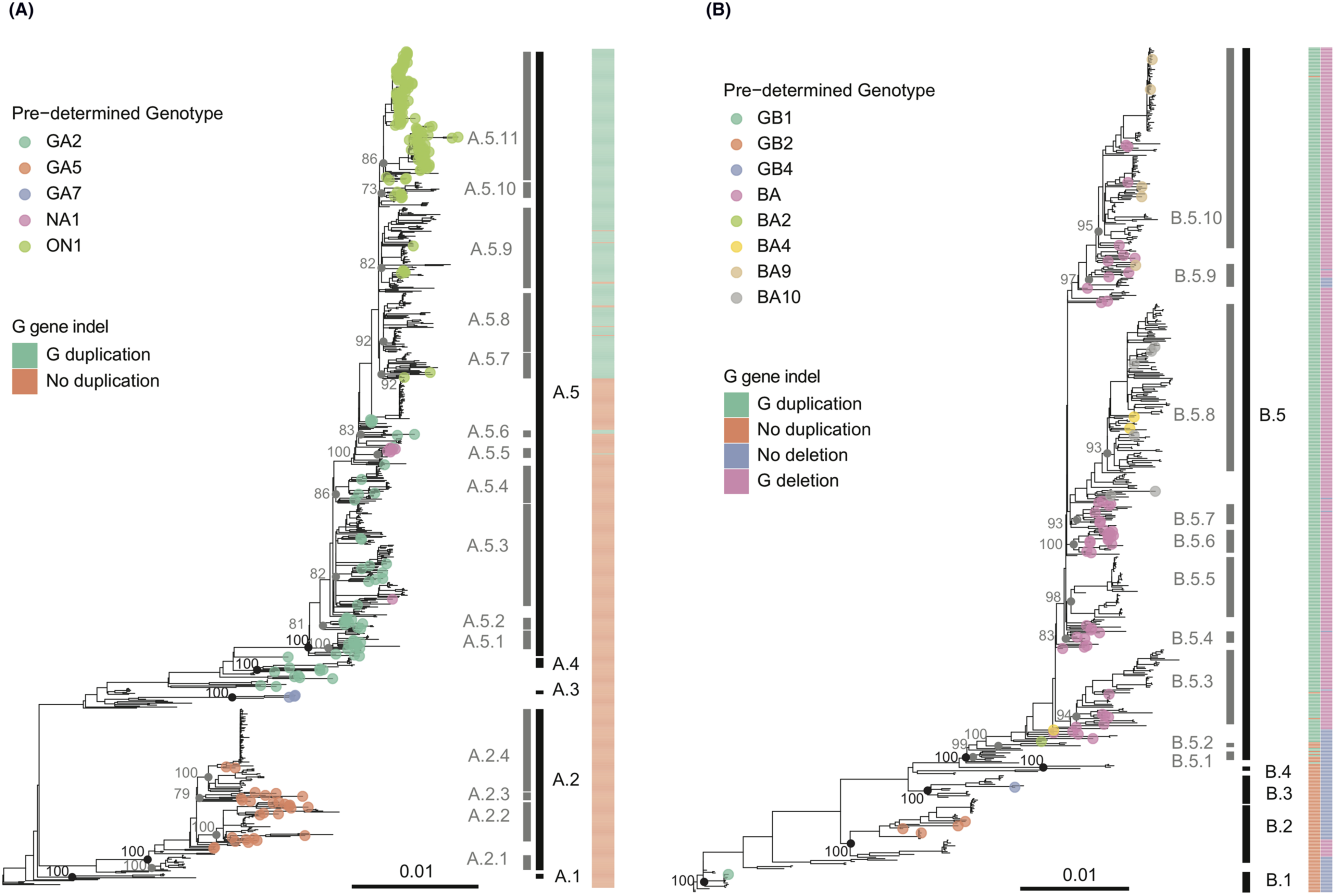
Maximum likelihood phylogeny of respiratory syncytial virus (RSV) RSV‐A (A) and RSV‐B (B) inferred from whole‐genome sequence (WGS) with the genotyping assignment. The genotype assignments are indicated with vertical black bars and are labeled on the right. The subclade assignments are indicated with vertical gray bars and are labeled on the left. Tip point colors represent the previously defined genotype names based on complete or partial G gene sequences. The nodes that define the genotype and subclade are indicated with black and gray node points, respectively. Bootstrap of each ancestral genotype/subclade node is marked. Colored columns on the right side represent G gene duplication and indels. Scale bars indicate 0.01 nucleotide substitution per site

With the use of this scheme, we identified 5 genotypes in RSV‐A (Figure 2A, Table 1). A.1 mainly contains the uncharacterized RSV‐A strains that are circulated in the past, A.2 contains the previously defined GA5 lineage, and 4 subclades denoted A.2.1–A.2.4 were identified. A.3 is mainly composed of viruses that are previously described as GA7 genotype. A.4 and A.5 contain the predominantly known global genotype GA2. A.5 has been subdivided into 11 descendant subclades (denoted A.5.1–A.5.11), and strains associated with the 72‐nt G gene duplication (ON) are found within genotype A.5.7–A.5.11. We categorized RSV‐B into 5 genotypes (Figure 2B, Table 1). B.1–B.3 contain previously described GB1, GB2, and GB4 genotype, respectively. B.4 genotype contains a relatively small group of sequences that have not been characterized before. B.5 contains the BA genotype, which consists of the 60‐nt duplication event in the G gene and is currently divided into 10 subclades.

**TABLE 1:** List of previously defined genotype name and detection time of new RSV genotype assignment.

Subtype	Genotype	Subclade	Detection time^a^	Previously defined genotype name^b^
A	A.1		1978–1998	
	A.2			
		A.2.1	1990–1994	
		A.2.2	2001–2015	GA5
		A.2.3	2001–2015	GA5
		A.2.4	1998–2013	GA5
	A.3		1984–1998	GA7
	A.4		1998–2009	GA2
	A.5			
		A.5.1	2007–2015	GA2
		A.5.2	2006–2010	GA2
		A.5.3	2008–2015	GA2, NA1
		A.5.4	2008–2015	GA2
		A.5.5	2011–2015	NA1
		A.5.6	2011–2015	GA2
		A.5.7	2012–2016	ON1
		A.5.8	2012–2016	
		A.5.9	2008–2016	
		A.5.10	2012–2017	ON1
		A.5.11	2011–2016	ON1
B	B.1		1979–1987	GB1
	B.2		1979–1991	GB2
	B.3		1989–2002	GB4
	B.4		2008–2012	
	B.5			
		B.5.1	1992–1996	
		B.5.2	1997–2013	
		B.5.3	2008–2015	BA
		B.5.4	2004–2009	BA
		B.5.5	2006–2012	
		B.5.6	2006–2015	BA
		B.5.7	2006–2013	BA
		B.5.8	2012–2016	BA
		B.5.9	2008–2013	BA
		B.5.10	2009–2016	BA

Abbreviation: RSV, respiratory syncytial virus.

^a^ Periods were detected up to 2017 and may underestimate the circulation time due to bias in GenBank deposition practices.

^b^ The previously defined genotype name was collected from GenBank.

### 3.3 Spatial and temporal distribution of RSV genotypes

**FIGURE 3:**
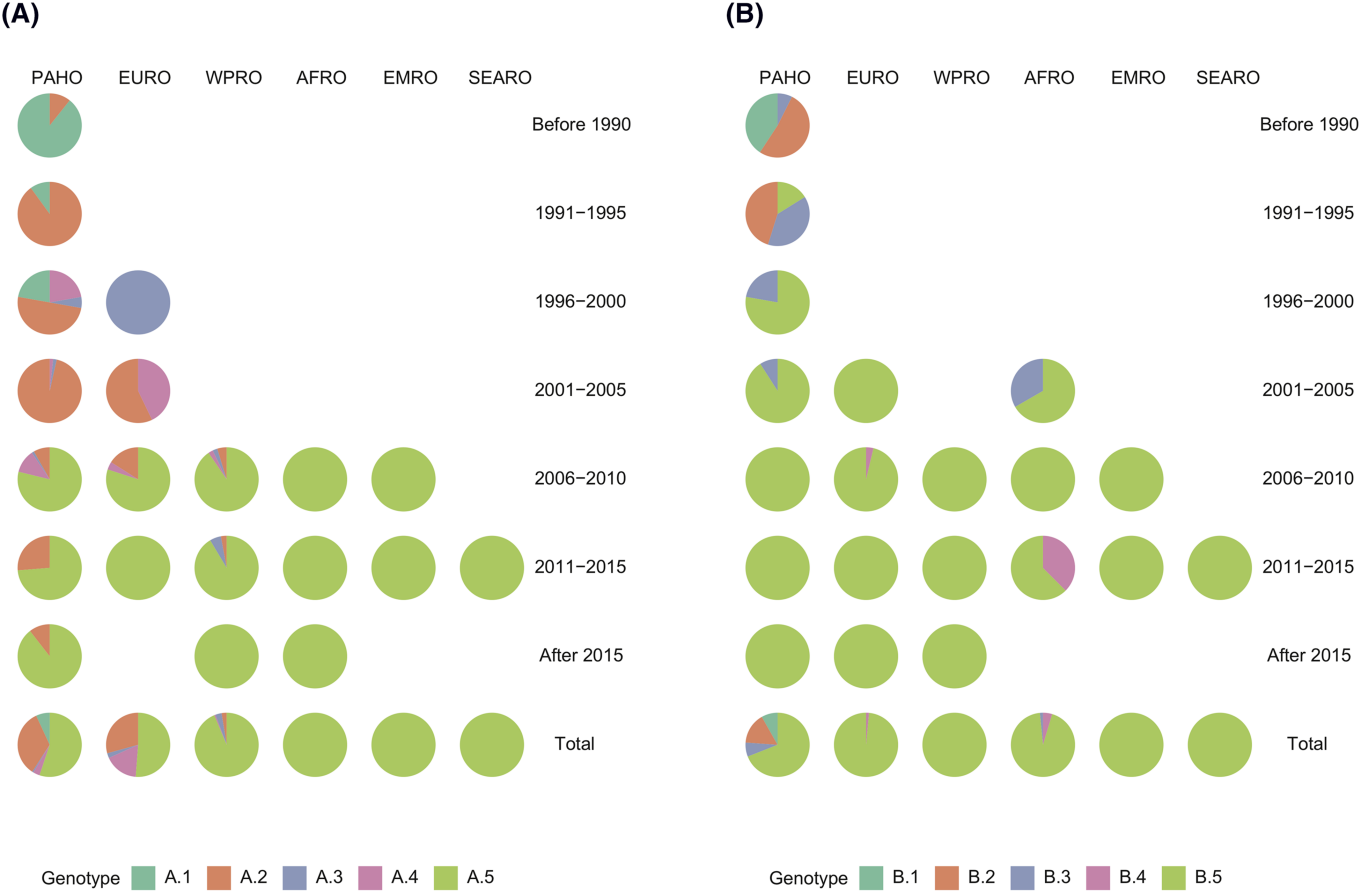
Spatial and temporal distribution of respiratory syncytial virus (RSV) RSV‐A (A) and RSV‐B (B) genotypes. The temporal and spatial distribution of RSV genotypes is based on the detection year and isolated WHO region of sequence for each assigned genotype. African Region (AFRO), Region of the Americas (PAHO), South‐East Asia Region (SEARO), European Region (EURO), Eastern Mediterranean Region (EMRO), and Western Pacific Region (WPRO)

We provide a description of the spatial and temporal distribution of RSV genotypes even though the bias in the samples sequenced does not provide a complete resolution of the past distribution of RSV variants. According to our revised genotyping system, RSV‐A had at least two important shifts in the dominant genotypes (Figure 3A). Before 1990, genotypes A.1 and A.2 that were isolated from the region of the Americas were the dominant genotypes. Since then, A.2 replaced these strains, became the dominant genotype, and co‐circulated with A.3 and A.4 in the American and European regions. Recently, a new genotype A.5 emerged and transmitted globally, but the previous dominant strains assigned as genotype A.2 were still circulating in some regions. Genotype B.1–B.3 were the dominant genotypes for RSV‐B in the past (Figure 3B). Post‐1995, the novel genotype B.5 emerged and became fixed in the population and has been circulating globally. B.4 is a group of strains that are detected after 2006. We have also deployed our genotyping assignment using the open‐source tools Nextstrain, which provides a graphical demonstration of the global transmission events and genomic diversity over time with our new RSV genotype assignment (https://nextstrain.org/community/JianiC/rsv-genotype).^30^

### 3.4 Automated RSV classification tool

Representative RSV genotypes were used to build a custom RSV classification module within label.^28^ The classifier ascribed the correct genotypes and subclades in all sequences but 49 instances, with 95.4% accuracy. Of these 49 sequences, 32 sequences from genotype B.2 were incorrectly assigned as genotype B.1, which are ancient RSV‐B strains circulating before 2000. The remaining 17 sequences were assigned to a sister clade (subclade) that shared ancestry with the correct genotype assignment (Tables S2 and S3). Overall, this classifier is fast and accurate in capturing our RSV classifications without requiring phylogenetic reconstruction.

## 4 DISCUSSION

In this study, we highlight the importance of WGS for RSV genotype assignment. We statistically compare the phylogeny derived from different RSV gene datasets with the likelihood score test and evolutionary rate estimation from different gene datasets. Our results indicate WGS should be used for genotype assignment. Our analysis is based on a recombination‐free dataset to avoid inferential biases. Twelve RSV sequences in our initial dataset showed some evidence of potential recombination. Because genomic recombination in RSV is believed to be extremely rare, it is most likely that these recombinants arose as a result of PCR or sequencing artifacts.^31^ Even though we did not observe a significant statistical difference in our analysis, the G gene duplication should be considered as a single biological event, and we implement an indel coding method to improve phylogenetic resolution.

There are several expectations for a widely acceptable RSV genotyping system. First, since more than 30 genotypes have been identified, we expect to have a reasonable number of RSV genotypes to simplify the study with different RSV strains. Secondly, genotype assignment should be able to capture the genetic diversity, thereby providing valuable insights into the ongoing evolution of the virus and playing an important role in its mitigation and control.^32^ Finally, we expect the novel emergent strains could be classified and easily added into the revised nomenclature system. Competing RSV genotype systems have been proposed (Table 2), including an influenza‐like system for RSV genotype classification based on the highest intra‐genotypic *p‐distance* as the minimum threshold to define a genotype,^14^, ^15^, ^33^ which are highly sensitive to sampling bias. Another recent systematic RSV genotype study attempted to use patristic distance (the shortest distance between two tips) instead of *p‐distance* to propose a new classification system.^16^ Both *p‐distance* and patristic distance are sensitive to the sequencing error and the length of sequences. In addition, a cutoff value of either genetic distance or patristic distance is always needed to standardize the molecular classification of RSV strains, which is likely to be problematic due to sampling bias in RSV, and delineation criteria may need to be reevaluated with continued surveillance of RSV strains.^15^, ^16^ We calculate pair‐wise node distance to assign genotype in our analysis. Our approach relies on the genetic divergence calculated from the tree tips to the most recent common ancestor for each genotype, which is less sensitive to the individual sequence quality and has advantages to the undersampled RSV sequences. In addition to the genetic differences between RSV genotypes, previous studies have suggested some RSV genotypes may have an advantage in transmission and circulation.^34^ Instead of identifying every lineage or strain, one of the major goals in this manuscript is to identify the group of strains that have the potential to circulate and to keep tracking them since their emergence. Therefore, we include the circulation time as a criterion to characterize the RSV strains that continue to circulate with a potential to be recognized as an emerging subclade. These are strains that may require elevation to genotype level with continued circulation. We expect new genotypes and subclades will be defined as RSV continues to circulate. The subclades identified are those strains among co‐circulating variants that are currently most likely to require monitoring effort. In addition, some reported genotypes may have an increased risk to cause severe symptoms,^35^ which is an important characterization of a classified genotype. However, these studies have limits that prevent consistent predictions to make firm conclusions about the potential clinical relevance of the different RSV genotypes. More information about the correlations between RSV strains and disease severity is needed to include these features in a genotyping system.

**TABLE 2:** Comparison of RSV molecular systematic proposals.

Reference	This study	Peret et al.^11^	Agoti et al.^14^	Goya et al.^15^	Ramaekers et al.^16^
Dataset	RSV whole‐genome sequence with isolation year in GenBank up to April 2019	G gene sequence in GenBank and sequences obtained in the laboratory	RSV G gene sequence isolated from 2006 through 2011 available in GenBank and sequences obtained in the laboratory	All RSV G ectodomain sequences available in GenBank up to February 2018	All RSV whole‐genome sequences available in GenBank up to January 2019
Genotyping region	CDS region of full‐length genome	Second hypervariable region of G ectodomain	G gene	G ectodomain	Full‐length genome
Genotyping criteria	Genotype: ≥70% bootstrap and maximal pair‐wise node distance within the clade (0.018 for RSV‐A and 0.024 for RSV‐B ); subclade: clade detection time ≥ 5 years within genotype	Genotype: ≥70% bootstrap; subtype: 96% nucleotide similarity within genotype	≥60% bootstrap and average genetic distance cutoff 1.5%	≥80% bootstrap and *P* distance ≥ 0.03 subs/site	≥70% bootstrap and patristic distance > 0.018 subs/site for RSV‐A, patristic distance > 0.026 subs/site for RSV‐B
Genotype name	A.1–A.5 (subclade: A.2.1–A.2.4, A.5.1–A.5.11); B.1–B.5 (subclade: B.5.1–B.5.10)	GA1–GA5 (22 subtypes among 5 genotypes); GB1–GB4 (6 subtypes among 4 genotypes)	GA1–GA7, SAA1; GB1–GB4, SAB1–SAB3, BA	GA1–GA3,;GB1–GB5	A1–A23; B1–B6

Abbreviation: RSV, respiratory syncytial virus.

It is crucial to share our updated genotype assignment so that new sequences can be easily added. We deploy our genotype assignment as well as the genotype assignment from previously published studies with Nextstrain, which allows a comparison and a continually updated visualization.^30^ We also provide a tool that enables the automated classification of newly generated RSV sequences. By using the platform provided, RSV sequences can be assigned with genotypes and subclades based on the similarity of the sequences that are included in our system.^28^ This fast and accurate RSV genotyping assignment tool will be valuable for the classification of novel sequences in future phylogenetic or diagnostic settings.

There are several limitations that need be addressed with any molecular systematic revision of RSV genotypes. In particular, RSV genotype assignment using WGS is subject to sampling bias. Only a limited number of sequences are currently available in GenBank, especially among older samples that were sequenced prior to the widespread and routine use of WGS, which may affect our genotype assignment. In addition, most samples sequenced were isolated from the regions in the Americas. With more RSV sequencing effort, we would expect the geographic distribution of sequence could be captured and effectively used for future updates to this genotype system.

In summary, we propose a revised RSV genotyping assignment that reflects the genetic diversity and circulation pattern of RSV. WGS should be used for future RSV genotyping revisions. In addition, the G gene duplication and other indels should be taken into account for the phylogenetic analysis as a single evolutionary event rather than multiple substitution patterns. Overall, a robust RSV genotype assignment based on WGS will greatly assist those working in clinical identification, epidemiological studies, and vaccine development.

## AUTHOR CONTRIBUTIONS

**Jiani Chen:** Formal analysis; project administration. **Xueting Qiu:** Conceptualization; methodology. **Vasanthi Avadhanula:** Conceptualization. **Samuel S. Shepard:** Software. **Do‐Kyun Kim:** Conceptualization. **James Hixson:** Conceptualization. **Pedro A. Piedra:** Conceptualization; funding acquisition. **Justin Bahl:** Conceptualization; funding acquisition; project administration; resources; supervision.

### PEER REVIEW

The peer review history for this article is available at https://publons.com/publon/10.1111/irv.12936.

## Supporting information


**Figure S1.** **Maximum likelihood phylogeny of RSV‐A (A) and RSV‐B (B) phylogeny inferred from WGS, L, G and F genes (from Left to right).** The color of the connected line between taxa indicates the isolated year for each strain. Scale bars indicate 0.01 nucleotide substitution per site.

**Figure S2.** **Criteria to assign genotypes and subgroups in RSV whole‐genome sequence phylogeny** (A) Number of genotypes to be assigned with different cutoff values of pair‐wise node distance. (B) Density distribution of clade circulation time (year) in RSV whole‐genome sequence phylogeny. Red dashed line indicates the 0.95 quantile of the distribution.

**Figure S3.** ***p‐distance* calculation within and between RSV genotypes.** A) RSV‐A intra‐genotypic and inter‐genotypic *p*‐distance for genotypes (left), subclades within genotype A.2 (middle) and genotype A.5 (right). B) RSV‐B intra‐genotypic and inter‐genotypic *p*‐distance for genotypes (left), subclades within genotype B.5 (right).

**Table S1:** Recombination events in the RSV WGS dataset.
**Table S2:** Accuracy of RSV genotype assignment tool.
**Table S3:** Accuracy of RSV genotype assignment tool with different test datasets.

**Data S1.** Supplementary Information.

## Data Availability

Data are publicly available on GenBank.
