# Supplementary figures and images for: Novel and extendable genotyping system for human respiratory syncytial virus based on whole‐genome sequence analysis
Source: Influenza Other Respir Viruses. 2021 Dec 10;16(3):492–500. doi: 10.1111/irv.12936 (PMC8983899; doi:10.1111/irv.12936)

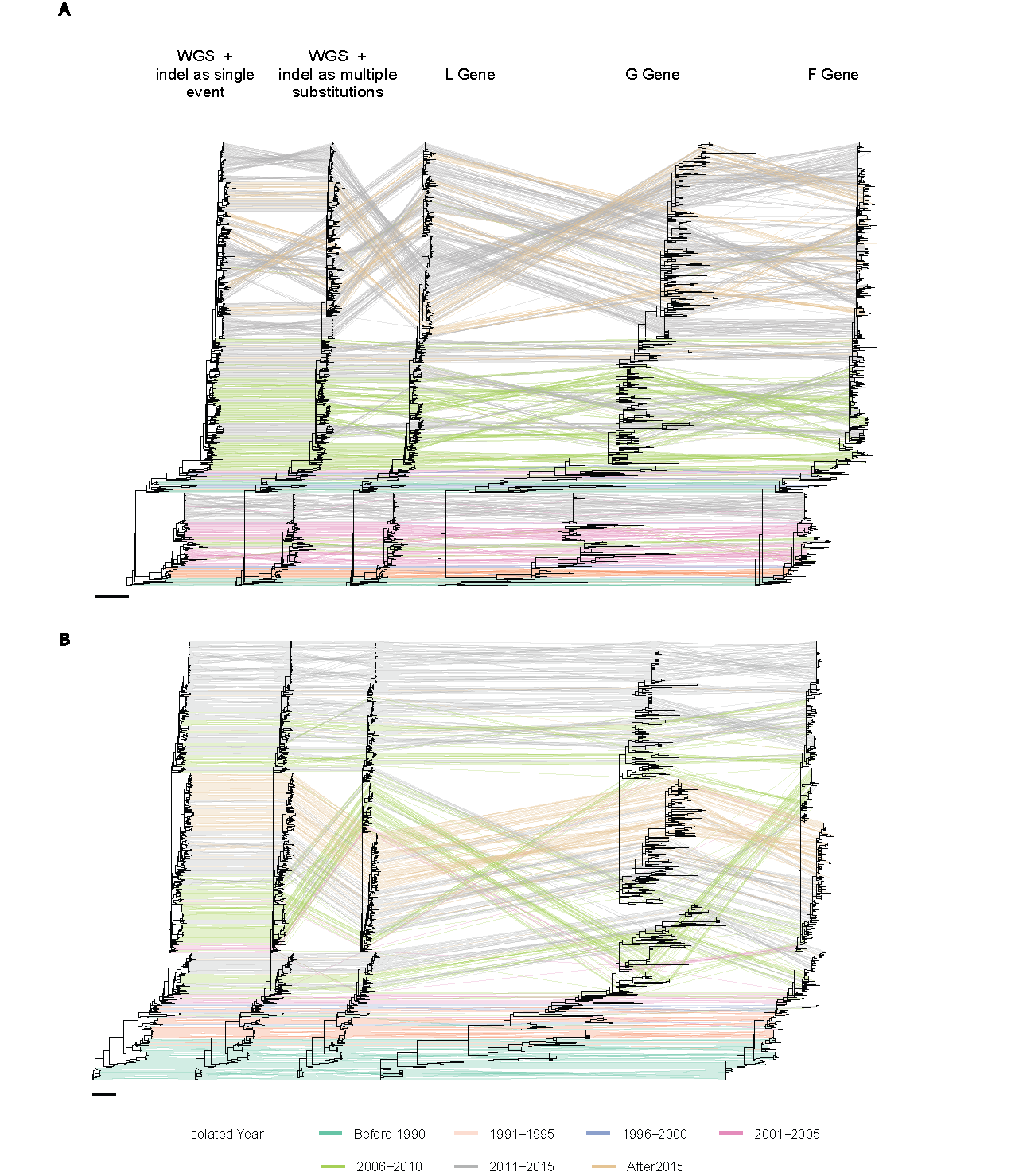

Supplement: Supplementary file 1 — Figure S1. Maximum likelihood phylogeny of RSV‐A (A) and RSV‐B (B) phylogeny inferred from WGS, L, G and F genes (from Left to right). The color of the connected line between taxa indicates the isolated year for each strain. Scale bars indicate 0.01 nucleotide substitution per site. [file IRV-16-492-s001.tif]

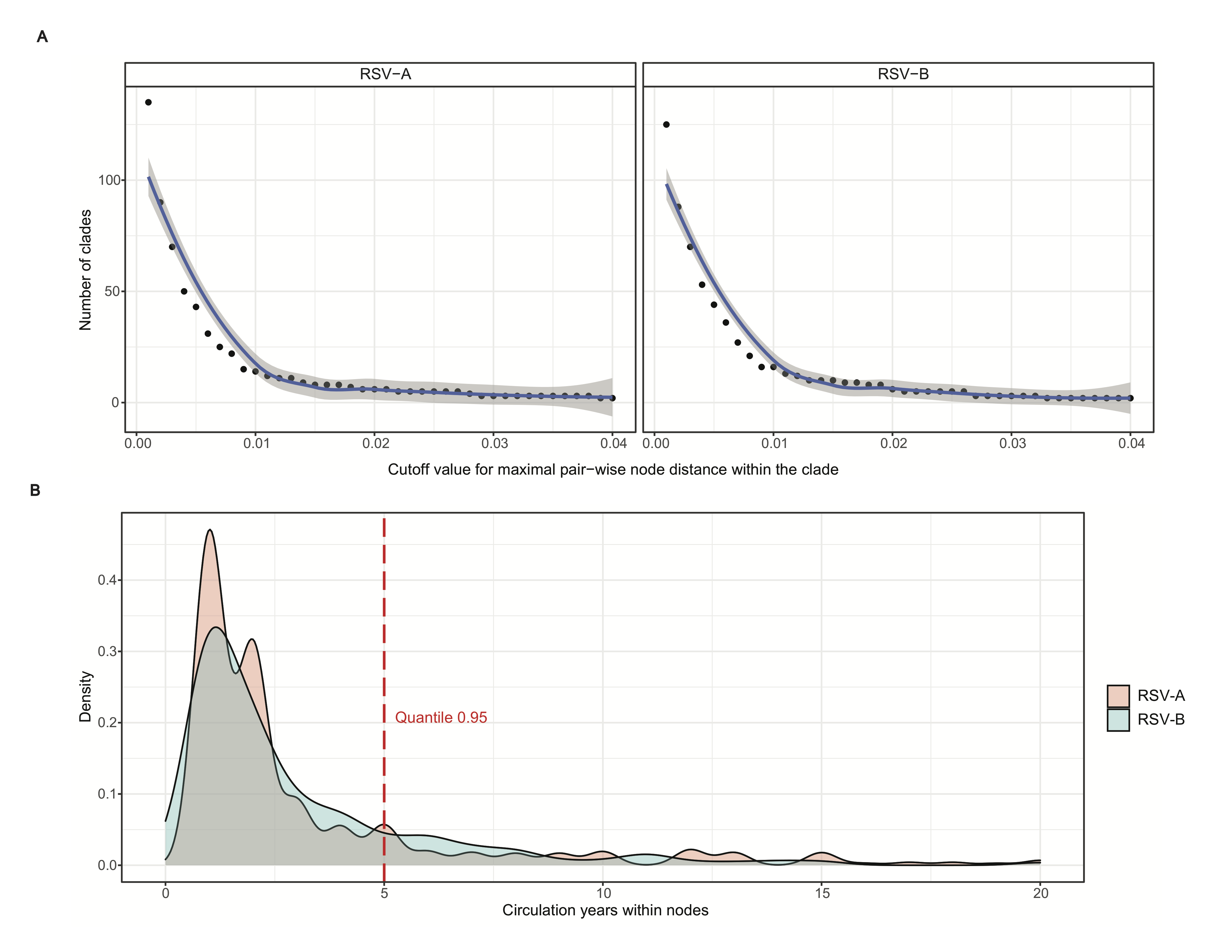

Supplement: Supplementary file 2 — Figure S2. Criteria to assign genotypes and subgroups in RSV whole‐genome sequence phylogeny (A) Number of genotypes to be assigned with different cutoff values of pair‐wise node distance. (B) Density distribution of clade circulation time (year) in RSV whole‐genome sequence phylogeny. Red dashed line indicates the 0.95 quantile of the distribution. [file IRV-16-492-s005.png]

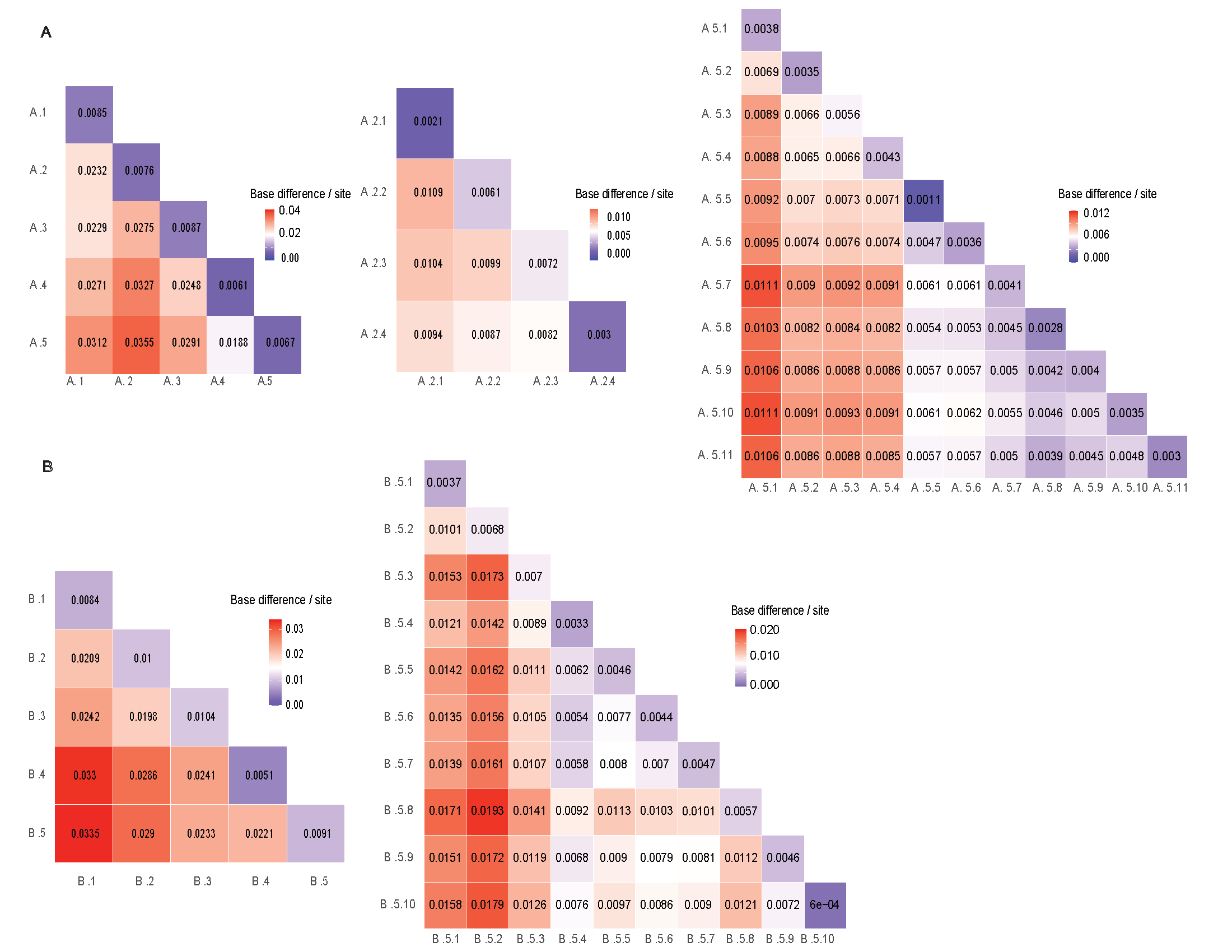

Supplement: Supplementary file 3 — Figure S3. p‐distance calculation within and between RSV genotypes. A) RSV‐A intra‐genotypic and inter‐genotypic p‐distance for genotypes (left), subclades within genotype A.2 (middle) and genotype A.5 (right). B) RSV‐B intra‐genotypic and inter‐genotypic p‐distance for genotypes (left), subclades within genotype B.5 (right). [file IRV-16-492-s002.png]
