# Supplementary material for: Novel and extendable genotyping system for human respiratory syncytial virus based on whole‐genome sequence analysis
Source: Influenza Other Respir Viruses. 2021 Dec 10;16(3):492–500. doi: 10.1111/irv.12936 (PMC8983899; doi:10.1111/irv.12936)
Supplement: Supplementary file 4 — Table S1: Recombination events in the RSV WGS dataset. Table S2: Accuracy of RSV genotype assignment tool. Table S3: Accuracy of RSV genotype assignment tool with different test datasets. [file IRV-16-492-s003.docx]

**Supplementary Methods**

**Genetic Distance Analysis**

To characterize the genetic diversity within and between genotypes, the average genetic distance within and between genotypes as well as subgroups were estimated from the alignments with the software MEGA X  using the most simplified method, *p‐distance*, which is the proportion of nucleotide sites at which two sequences being compared were different.

**Supplementary Table 1: Recombination events in the RSV WGS dataset.**

| **Subtype** | **Recombinant** | **Minor Parent** | **Major Parent** | **Start in Alignment** | **End in Alignment** | **Best P-Value** | **Method** |
| --- | --- | --- | --- | --- | --- | --- | --- |
| A | JX015495 | JX015481 | KT285064 | 6374 | 12817 | 1.48E-06 | MaxChi |
|  | JX069800 | KP119746 | MG642026 | 12247 | 12368 | 0.034705806 | RDP |
|  | JX627336 | KF826830 | KX765941 | 543 | 1064 | 0.032280115 | RDP |
|  | KJ672480 | MG642074 | KU950480 | 4222 | 4302 | 2.75E-08 | GENECONV |
|  | KJ672482 | JX069800 | MH760612 | 12254 | 12294 | 0.030458888 | GENECONV |
|  | MF001054 | JQ901456 | MF001052 | 1951 | 2024 | 8.31E-14 | RDP |
| B | KJ627251 | KJ627342 | KJ627254 | 9522 | 13303 | 8.82E-10 | RDP |
|  | KJ672473 | LC385000 | KJ672481 | 9522 | 13303 | 0.000338187 | RDP |
|  | KJ939932 | Unknown | KJ939934 | 3742 | 6407 | 6.63E-07 | RDP |
|  | KJ939933 | KJ939931 | MH760701 | 3959 | 6516 | 5.08E-09 | SiSscan |
|  | KY249663 | KY249674 | KY249669 | 3490 | 4262 | 1.33E-10 | RDP |
|  | KY924878 | MH760677 | Unknown | 5544 | 13586 | 1.04E-09 | Maxchi |

**Supplementary Table 2: Accuracy of RSV genotype assignment tool**

| **Genotype** | **Subclade** | **Annotation ^a^** | **Sequences for training module** | **Classification tool ^b^** | **Accuracy (%) ^c^** |
| --- | --- | --- | --- | --- | --- |
| A.1 |  | 6 | 6 | 6 | 100.00% |
| A.2 |  | 151 | 65 | 151 | 100.00% |
|  | A.2.1 | 16 | 16 | 16 | 100.00% |
|  | A.2.2 | 41 | 20 | 41 | 100.00% |
|  | A.2.3 | 9 | 9 | 9 | 100.00% |
|  | A.2.4 | 85 | 20 | 85 | 100.00% |
| A.3 |  | 6 | 6 | 6 | 100.00% |
| A.4 |  | 15 | 15 | 15 | 100.00% |
| A.5 |  | 516 | 196 | 516 | 100.00% |
|  | A.5.1 | 20 | 20 | 20 | 100.00% |
|  | A.5.2 | 13 | 13 | 13 | 100.00% |
|  | A.5.3 | 105 | 20 | 105 | 100.00% |
|  | A.5.4 | 39 | 20 | 38 | 97.40% |
|  | A.5.5 | 11 | 11 | 10 | 90.90% |
|  | A.5.6 | 8 | 8 | 4 | 50.00% |
|  | A.5.7 | 27 | 27 | 27 | 100.00% |
|  | A.5.8 | 61 | 20 | 57 | 93.40% |
|  | A.5.9 | 83 | 20 | 83 | 100.00% |
|  | A.5.10 | 17 | 17 | 17 | 100.00% |
|  | A.5.11 | 132 | 20 | 120 | 90.90% |
| B.1 |  | 11 | 11 | 11 | 100.00% |
| B.2 |  | 42 | 20 | 10 | 23.81% |
| B.3 |  | 16 | 16 | 16 | 100.00% |
| B.4 |  | 4 | 4 | 4 | 100.00% |
| B.5 |  | 424 | 149 | 419 | 98.80% |
|  | B.5.1 | 7 | 7 | 7 | 100.00% |
|  | B.5.2 | 4 | 4 | 4 | 100.00% |
|  | B.5.3 | 53 | 20 | 53 | 100.00% |
|  | B.5.4 | 9 | 9 | 9 | 100.00% |
|  | B.5.5 | 43 | 20 | 43 | 97.70% |
|  | B.5.6 | 17 | 17 | 17 | 100.00% |
|  | B.5.7 | 15 | 15 | 14 | 93.30% |
|  | B.5.8 | 118 | 20 | 118 | 100.00% |
|  | B.5.9 | 17 | 17 | 17 | 100.00% |
|  | B.5.10 | 141 | 20 | 141 | 100.00% |
| Total |  | 1202 | 477 | 1147 | 95.42% |

1. The number of sequences in genotype or subclade
2. The number of sequences that are assigned to the correct genotype or subclade using classification tool
3. The percentage of sequences that are assigned to the correct genotype or subclade using classification tool

**Supplementary Table 3: Accuracy of RSV genotype assignment tool with different test datasets**

| **Test Data** | **Sequence Count** | **Minimum sequence length (nt)** | **Maximal sequence length (nt)** | **Accuracy ^a^** |
| --- | --- | --- | --- | --- |
| SVM training set  (self-validation) | 1176 | 14902 | 15333 | 95.4% |
| RSV full length sequences submitted after April, 2019 | 582 | 14906 | 15276 | 100% |
| G gene sequences from SVM training set | 1176 | 896 | 970 | 91.8% |

1. Accuracy is evaluated by the number of sequences that are correctly annotated over the number of test sequences.

**Supplementary Figure Legends**

**Supplementary Figure 1. Maximum likelihood phylogeny of RSV-A (A) and RSV-B (B) phylogeny inferred from WGS, L, G and F genes (from Left to right).** The color of the connected line between taxa indicates the isolated year for each strain. Scale bars indicate 0.01 nucleotide substitution per site.

**Supplementary Figure 2. Criteria to assign genotypes and subgroups in RSV whole-genome sequence phylogeny** (A) Number of genotypes to be assigned with different cutoff values of pair-wise node distance. (B) Density distribution of clade circulation time (year) in RSV whole-genome sequence phylogeny. Red dashed line indicates the 0.95 quantile of the distribution.

**Supplementary Figure 3. *p-distance* calculation within and between RSV genotypes.** A) RSV-A intra-genotypic and inter-genotypic *p*-distance for genotypes (left), subclades within genotype A.2 (middle) and genotype A.5 (right). B) RSV-B intra-genotypic and inter-genotypic *p*-distance for genotypes (left), subclades within genotype B.5 (right).
